# Testing the efficiency of plant artificial microRNAs by transient expression in *Nicotiana benthamiana* reveals additional action at the translational level

**DOI:** 10.3389/fpls.2014.00622

**Published:** 2014-11-19

**Authors:** Shi Yu, Guillaume Pilot

**Affiliations:** Department of Plant Pathology Physiology and Weed Science, Virginia Polytechnic Institute and State UniversityBlacksburg, VA, USA

**Keywords:** Arabidopsis, *Nicotiana benthamiana*, artificial microRNA, translational inhibition, mRNA stability, transient expression

## Abstract

Artificial microRNAs (amiRNAs) have become an important tool to assess gene functions due to their high efficiency and specificity to decrease target gene expression. Based on the observed degree of complementarity between microRNAs (miRNAs) and their targets, it was widely accepted that plant miRNAs act at the mRNA stability level, while the animal miRNAs act at the translational level. Contrary to these canonical dogmas, recent evidence suggests that both plant and animal miRNAs act at both levels. Nevertheless, it is still impossible to predict the effect of an artificial miRNA on the stability or translation of the target mRNA in plants. Consequently, identifying and discarding inefficient amiRNAs prior to stable plant transformation would help getting suppressed mutants faster and at reduced cost. We designed and tested a method using transient expression of amiRNAs and the corresponding target genes in *Nicotiana benthamiana* leaves to test the efficacy of amiRNAs for suppression of the target protein accumulation. The ability of the amiRNAs to suppress the target gene expression in *N. benthamiana* was then compared to that in stably transformed Arabidopsis. It was found that the efficacy of 16 amiRNAs, targeting a total of four genes, varied greatly. The effects of amiRNAs on target mRNA accumulation did not always correlate with target protein accumulation or the corresponding phenotypes, while a similar trend of the silencing efficacy of amiRNAs could be observed between *N. benthamiana* and stably transformed Arabidopsis. Our results showed that, similar to endogenous plant miRNAs, plant amiRNAs could act at the translational level, a property needed to be taken into account when testing the efficacy of individual amiRNAs. Preliminary tests in *N. benthamiana* can help determine which amiRNA would be the most likely to suppress target gene expression in stably transformed plants.

## Introduction

MicroRNAs (miRNAs) are a class of small noncoding RNAs of 20–24 nucleotides in length that regulate target gene expression at the post-transcriptional level in eukaryotes (Brodersen and Voinnet, 2009; Rogers and Chen, 2013). miRNAs are processed from longer precursor transcripts to a stable hairpin structure with two complementary short RNA strands, which are further processed to miRNA:miRNA^*^ duplexes, by RNaseIII enzymes (miRNA^*^ being the passenger strand). The miRNA:miRNA^*^ duplexes are then transported out of the nucleus to the cytoplasm, where the miRNA^*^ are degraded. Mature miRNAs are bound by ARGONAUTE proteins to form the RNA-induced silencing complex (RISC). miRNAs serve as guides for RISC to bind target mRNA(s) and silence gene expression (Brodersen and Voinnet, 2009; Meng et al., 2011; Sun, 2012). It is believed that perfect or near-perfect complementarity favors RISC-catalyzed endonucleolytic mRNA cleavage, while central mismatches promote translational repression (Brodersen and Voinnet, 2009; Huntzinger and Izaurralde, 2011). In contrast to most animal miRNAs, most plant miRNAs show perfect or near-perfect complementarity to their targets, hence mRNA cleavage is deemed to be the dominant mode of action in plants (Brodersen and Voinnet, 2009; Huntzinger and Izaurralde, 2011). In disagreement with this postulate, evidence from recent reports suggests that translational repression plays a vital role in regulating target gene expression in plants (Aukerman and Sakai, 2003; Chen, 2004; Gandikota et al., 2007; Brodersen et al., 2008; Dugas and Bartel, 2008; Lanet et al., 2009; Zhu et al., 2009; Beauclair et al., 2010; Zhu and Helliwell, 2011; Alonso-Peral et al., 2012; Li et al., 2013b; Ma et al., 2013; Meijer et al., 2013). It has also recently been shown that miRNAs affect target gene DNA methylation in plants (Wu et al., 2010, 2012). Thus, the action mode of miRNAs on gene expression appears more diverse than initially thought, with effects on target mRNA cleavage, translation inhibition and DNA methylation, possibly exerted concomitantly (Pillai et al., 2007; Voinnet, 2009).

Plant artificial microRNAs (amiRNAs) are produced by expression of a miRNA gene genetically modified by replacing the original miRNA:miRNA^*^ duplex region with customized sequences to silence one or more genes of interest in various plant species (Schwab et al., 2006; Ossowski et al., 2008). Plant amiRNA technology utilizes the high degree of complementarity between the miRNAs and their mRNA targets to ensure the silencing specificity for the amiRNAs. Compared to RNA interference (RNAi) and virus-induced gene silencing (VIGS), plant amiRNAs have several advantages such as minimal predicted off-target effects and ability for multigene silencing (Schwab et al., 2006; Ossowski et al., 2008). Use of amiRNA is also a potential strategy for engineering plant resistance to viruses (Sablok et al., 2011; Jelly et al., 2012). The Web-based amiRNA designer, developed by the Weigel lab, (WMD; Ossowski et al., personal communication) provides a platform to design gene-specific amiRNA candidates for more than 100 plant species. While the candidates are ranked by an algorithm according to the canonical sequence complementarity and hybridization energy, WMD does not consider factors that may elicit translational repression, such as target mRNA structure and mRNA binding proteins (Schwab et al., 2005; Fabian et al., 2010; Pasquinelli, 2012). The evidence that translational control plays an important part in the effect of endogenous plant miRNAs questions the validity of the prediction for a given amiRNA to silence its target gene solely based on the cleavage efficiency, often measured by decreases in target mRNA abundance. The possible variation in efficacy between different amiRNA candidates to the same target gene might thus hinder the broad application of amiRNA technology for gene silencing. Two articles reported the testing of amiRNAs by co-expression of the tagged target gene and the candidate amiRNAs in Arabidopsis protoplasts (Kim and Somers, 2010; Li et al., 2013a). Interestingly, the authors noticed that, similar to miRNAs, many amiRNAs exert translational inhibition of the target gene while leading to a small decrease in the target mRNA abundance.

Our previous work with amiRNAs targeting the *LOSS OF GDU 2* (*LOG2*, AT3G09770) gene showed that these amiRNAs led to a reduction of less than 80% of *LOG2* mRNA content while effectively triggering a LOG2-dependent phenotype (Pratelli et al., 2012). Here, we report a more in depth analysis of the effect of these four amiRNAs on the *LOG2* mRNA and protein accumulation in both Arabidopsis and *Nicotiana benthamiana*. To expand our analysis, the effects of twelve amiRNAs targeting three additional genes were studied. While assays aiming at testing amiRNA efficacy have been developed, they rely on the use of Arabidopsis protoplasts (Kim and Somers, 2010; Li et al., 2013a), a technique not available in every laboratory. Thus, we tested whether *N. benthamiana* could be substituted to Arabidopsis protoplasts for testing amiRNA efficacy, and compared the results obtained by expression of the amiRNAs in *N. benthamiana* and Arabidopsis.

## Results

### Analysis of amiRNAs targeting *LOG2*

**Figure 1:**
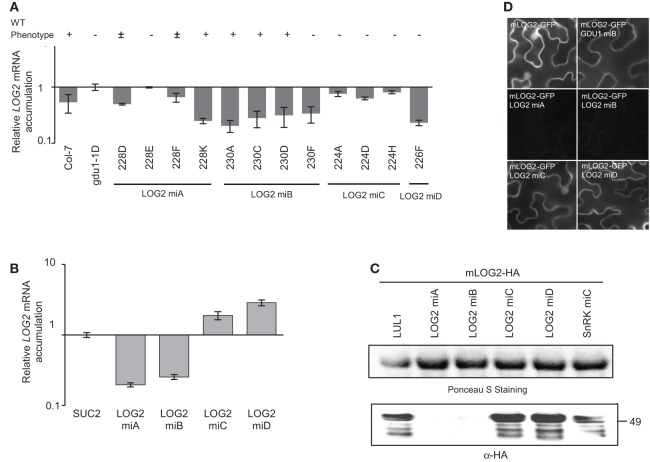
**Effects of artificial miRNAs targeting *LOG2* in stably transformed Arabidopsis and in transiently transformed *N. benthamiana*. (A)** amiRNAs targeting *LOG2* (LOG2 miA, miB, miC and miD) were stably introduced into the *gdu1-1D* Arabidopsis mutant. The phenotype of about 20 progenies of each transformant line was recorded (“+,” wild type; “±,” intermediate between Gdu1D and WT; “−,” Gdu1D). mRNA level of *LOG2* in each line was measured by qRT-PCR, normalized with *ACT2* and expressed relative to the level in *gdu1-1D*. **(B)** Relative *LOG2* mRNA levels in transiently transformed *N. benthamiana*. *mLOG2* (ubiquitination inactive LOG2) was co-expressed with *SUC2* or amiRNA^LOG2^ (see main text). *LOG2* mRNA levels were measured by qRT-PCR and expressed relative to levels in leaves transformed with *mLOG2* and *SUC2*. Co-expression of *SUC2* was used as co-expression control. **(C)** Western blot detection of the accumulation of the mLOG2 protein (ubiquitination inactive LOG2), when co-expressed transiently with four amiRNA^LOG2^ in *N. benthamiana*. amiRNA^SnRK1.1^-C (SnRK miC) was used as non-targeting amiRNA negative control. Numbers on the right indicate molecular weight in kDa. Co-expression of LUL1 (see main text) was used as co-expression control. **(D)** Analysis of amiRNAs effect on mLOG2-GFP fluorescence in transiently transformed *N. benthamiana* epidermis cells. Fluorescence microscope pictures were all taken with the same settings. Error bars represent standard error of at least three biological replicates.

In an earlier report, we found that the LOG2 ubiquitin ligase interacted with the GLUTAMINE DUMPER 1 (GDU1) protein, and that knockdown or knockout of *LOG2* in Arabidopsis suppressed the phenotype caused by the over-expression of *GDU1* (Pratelli et al., 2012). *GDU1* over-expression, as in the *gdu1-1D* mutant, leads to reduced-size plants secreting glutamine at the leaf margin, and displaying curled and darker green leaves in addition to several metabolic phenotypes (Gdu1D phenotype, Pilot et al., 2004). Expression in *gdu1-1D* of amiRNA^LOG2^-A and -B targeting *LOG2* suppressed endogenous *LOG2* gene activity (three out of 24 and six out of 21 transformants, respectively): a wild type phenotype indicates a suppression of *LOG2* activity and the Gdu1D phenotype indicates a lack of suppression (Figure 1A). In none of the lines, wild type phenotype was due to loss of GDU1 over-expression (Figure S1A). Some lines displayed an intermediary phenotype, probably due to partial suppression of the *LOG2* gene activity (Figure S2). In these lines, *LOG2* mRNA accumulation was reduced by less than 80% in the 35S-GDU1-cMyc (Pratelli et al., 2012) or *gdu1-1D* (Figure 1A) backgrounds, while the strength of wild type phenotype (suppression of *LOG2* activity) did not directly correlate with the reduction in *LOG2* mRNA accumulation (compare lines 230D and 230F; Figure 1A).

Two amiRNAs (amiRNA^LOG2^-C and -D) could not lead to loss of the Gdu1D phenotype in any of 22 and 21 independent transformants, respectively, suggesting that these amiRNAs are not as efficient as amiRNA^LOG2^-A and -B to suppress *LOG2* expression (Figure 1A). This hypothesis was tested by transiently co-expressing *mLOG2* (ubiquitination defective LOG2, fused to the HA tag or the GFP) and the four amiRNAs in *N. benthamiana* and testing for *LOG2* expression, both at the mRNA and protein levels. *Agrobacterium*-mediated transient expression in leaves of *N. benthamiana* is a widely used technique to study proteins (e.g., Voinnet et al., 2003; Popescu et al., 2007; Liu et al., 2010; Shah et al., 2013), and has been recently used for expressing and testing miRNAs and amiRNAs (Bhagwat et al., 2013; Liu et al., 2014). The control for this experiment consisted in the co-expression of the target protein mLOG2-HA with LUL1, a LOG2 paralog (Pratelli et al., 2012), to establish mLOG2-HA protein accumulation baseline when co-expressed with an unrelated construct. Co-expression of the unrelated amiRNA^SnRK1.1^-C (see below) showed that the effect on mLOG2-HA protein is specific to the amiRNA targeting this transgene (Figure 1C). *mLOG2* mRNA levels were reduced by 75–80% by co-expression with amiRNA^LOG2^-A and -B (Figure 1B), while no mLOG2 protein accumulation could be detected (Figure 1C). In contrast, amiRNA^LOG2^-C and -D slightly increased *mLOG2* mRNA and protein contents (Figures 1B,C). Observing the fluorescence of mLOG2-GFP co-expressed with amiRNA^LOG2^ confirmed that amiRNA^LOG2^-A and -B were the most efficient at reducing the accumulation of mLOG2 protein (Figure 1D).

These data indicate that amiRNA^LOG2^-C and -D could not suppress LOG2 protein accumulation in *N. benthamiana*, and were unable to suppress the Gdu1D phonotype in Arabidopsis. In Arabidopsis, amiRNA^LOG2^-A and -B were able to suppress the Gdu1D phenotype similar to *LOG2* knock-out lines, suggesting that *LOG2* expression is suppressed, but with still 20–25% of *LOG2* mRNA present in the cells. These data suggest that amiRNA^LOG2^-A and -B also act at the translational level to suppress *LOG2* expression.

### Design of an approach to compare amiRNA efficiency in arabidopsis and *N. benthamiana*

Based on the encouraging correlation of the results in Arabidopsis and *N. benthamiana*, we decided to develop an approach to help determining the efficiency of amiRNAs to suppress the expression of their target genes before Arabidopsis transformation and plantlet selection. We extended this analysis to three additional genes, to parallel the article from Li et al. (2013a), but instead of expressing the amiRNAs and their target genes in Arabidopsis protoplasts, we tested the co-expression in *N. benthamiana* leaves. We further tested the reliability of the prediction of amiRNA efficacy from transient expression assays into stably transformed Arabidopsis.

Three genes (*GDU1*, AT4G31730; *SnRK1.1*, AT3G01090; and *MIPS1*, AT4G39800) were selected as amiRNA targets because of interests in our laboratory or alteration of the expression of these genes had been shown to lead to phenotypic changes. Suppression of *MIPS1* reduces inositol synthesis and causes spontaneous cell death (Donahue et al., 2010). Overexpression of *SnRK1.1* in Landsberg *erecta* leads to glucose sensitivity, late flowering and delayed senescence (Baena-Gonzalez et al., 2007). For each of these three genes, four amiRNAs targeting different regions within the genes were designed using WMD (http://wmd3.weigelworld.org/): the criteria were that they should bind different regions of the target mRNAs and displayed the best scores (Figures S3, S4; Table S2).

amiRNA constructs were introduced into Arabidopsis lines expressing the corresponding target genes cloned in frame with the HA tag, cMyc tag or the GFP coding sequences, to ease protein detection (Table S1). These constructs are under the control of the viral CaMV 35S promoter, and expressing the amiRNA under the control of the CaMV 35S promoter would likely trigger suppression (Daxinger et al., 2008; Pilot, unpublished results). The amiRNAs were thus expressed under the control of another viral promoter, the CsVMV (Verdaguer et al., 1998), which has been shown to be compatible with the CaMV 35S promoter (see above, and Pratelli et al., 2012, for LOG2). Phenotypes of the transformants were recorded, and both target mRNA and protein levels were measured in the progenies.

In parallel, the amiRNAs were transiently co-expressed in *N. benthamiana* leaves with the target genes fused to the coding sequence of protein tags, and the corresponding target mRNA and protein levels were assessed. The viral RNAi inhibitor p19 has been shown to enhance protein production in *N. benthamiana* (Voinnet et al., 2003), and is routinely used to express genes in this system because it prevents suppression of the expression of the transgene. While Ahn et al. suggested that p19 would not affect miRNA-mediated silencing (Ahn et al., 2011), we found that p19 strongly suppressed the amiRNA^LOG2^-A and -B-induced silencing in *N. benthamiana* (data not shown). Yu et al. also showed that miRNA methylation, which is a critical step in miRNA biogenesis, is interfered by RNA silencing suppressor (Yu et al., 2005, 2006). Consequently, all assays in *N. benthamiana* were performed without co-infiltration of p19 and measuring target protein and mRNA content 2 days after infiltration, to prevent any silencing of the constructs. The first control for these experiments corresponded to a co-expression of the target construct with a construct available in the laboratory encoding an unrelated protein under the control of CsVMV promoter: LOG2-HA; LUL1 (AT5G03200), and LUL4-HA (AT5G19080), paralogs of LOG2; the sucrose transporter SUC2 (AT1G22710). The second control was an amiRNA targeting another gene from the present study (amiRNA^SnRK1.1^-C and amiRNA^LOG2^-B). These controls ensured that the expression of the amiRNAs did not perturb protein accumulation and that the effect of the amiRNA is specific to the target gene. We finally compared the results of transient expression in *N. benthamiana* with stable expression in Arabidopsis. The entire procedure is depicted in Figure S5.

### Comparison of the effect of amiRNAs to suppress the expression of *MIPS1*

**Figure 2:**
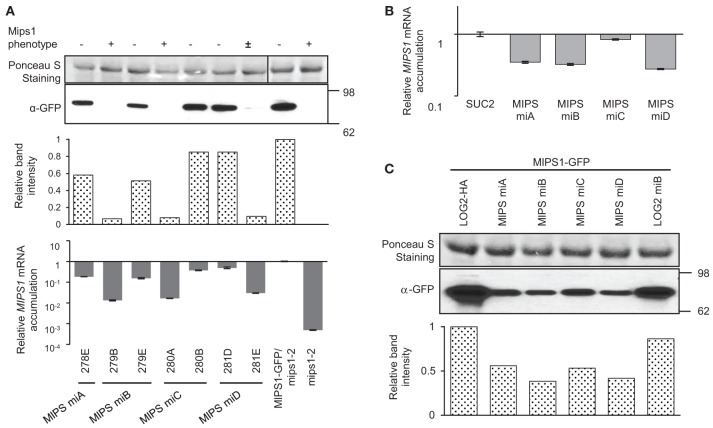
**Analysis of amiRNAs targeting *MIPS1* in stably transformed Arabidopsis and in transiently transformed *N. benthamiana*. (A)** amiRNAs targeting *MIPS1* (MIPS miA, miB, miC and miD) were stably introduced into the MIPS1-GFP/*mips1-2* background. The phenotype of about 10 progenies of each line was recorded (“−,” wild type; “±,” intermediate between *mips1* and WT; “+”, *mips1*). MIPS1 protein levels were determined by western blot with an anti-GFP antibody; band intensities are expressed relative to the maximum (line MIPS1-GFP). *MIPS1* relative mRNA levels were measured by qRT-PCR (accumulation is expressed relative to the mRNA levels in MIPS1-GFP/*mips1-2*). Error bars represent standard error processed by qbase^PLUS^. **(B)** Relative *MIPS1* mRNA levels in transiently transformed *N. benthamiana*. *MIPS1* was co-expressed with *SUC2* or amiRNA^MIPS1^. *MIPS1* mRNA levels were estimated by qRT-PCR and expressed relative to levels in leaves transformed with *MIPS1* and *SUC2*. Error bars represent standard error of three biological replicates. **(C)** Western blot showing the effects of amiRNA^MIPS1^ on MIPS1 protein accumulation in transiently transformed *N. benthamiana;* band intensities are expressed relative to the maximum (sample LOG2-HA). amiRNA^LOG2^ (LOG2 miB) was used as negative control, and co-expression of LOG2-HA was used as co-expression control. Numbers on the right indicate molecular weight in kDa.

Four different amiRNA^MIPS1^ were introduced into a 35S-MIPS1-GFP/*mips1-2* Arabidopsis line (provided by Dr. G. Gillaspy). For each amiRNA, about 20 transformants were selected and screened for lower MIPS1-GFP fluorescence at the seedling stage. Five transformants that showed lowest GFP fluorescence (highest suppression of *MIPS1* expression) were transferred to soil and their seeds were collected. GFP fluorescence of the T2 plants was screened again at the seedling stage, and, for each amiRNA, six progenies of two lines that showed the strongest reduction in GFP fluorescence were transferred to soil and used for the study of target mRNA and protein levels. A total of seven transformant lines were studied for *MIPS1*, corresponding to the four amiRNAs (plant sample for line 278D was lost). Two of these seven lines (279B and 280A) showed a phenotype similar to the *mips1-2* mutant in the T2 generation, and one (281E) showed an intermediate phenotype, despite the fact that they all displayed the lowest MIPS1-GFP fluorescence during the screening (Figure 2A and Figure S6). Lines 279B, 280A, and 281E accumulated less *MIPS1* transcript and MIPS1-GFP protein was reduced by more than 90%, contrary to the other lines (Figure 2A). Lines 278E, 279E, 280B, and 281D showed little reduction in *MIPS1* mRNA and protein accumulation, and displayed the wild type phenotype. Overall, the four different amiRNA^MIPS1^ could lead to similar efficiency to silence *MIPS1* gene, and MIPS1 protein accumulation correlated to *MIPS1* mRNA accumulation and the phenotype.

The efficacy of amiRNA^MIPS1^ was estimated by transient assay in *N. benthamiana* leaves. In this system, expression of amiRNA^MIPS1^-A, -B and -D reduced *MIPS1* transcript abundance by 65–75%, less than in Arabidopsis (Figure 2B), while amiRNA^MIPS1^-C could not reduce *MIPS1* mRNA level by more than 20%. In parallel, the reduction of MIPS1 protein accumulation was moderate (~50%) and similar for the four amiRNA^MIPS1^ (Figure 2C). The effects of the various amiRNA^MIPS1^ at the target mRNA and the protein levels were different, with amiRNA^MIPS1^-C being potent at reducing MIPS1 protein but not *MIPS1* mRNA accumulation.

### Comparison of the effect of amiRNAs to suppress the expression of *SnRK1.1*

**Figure 3:**
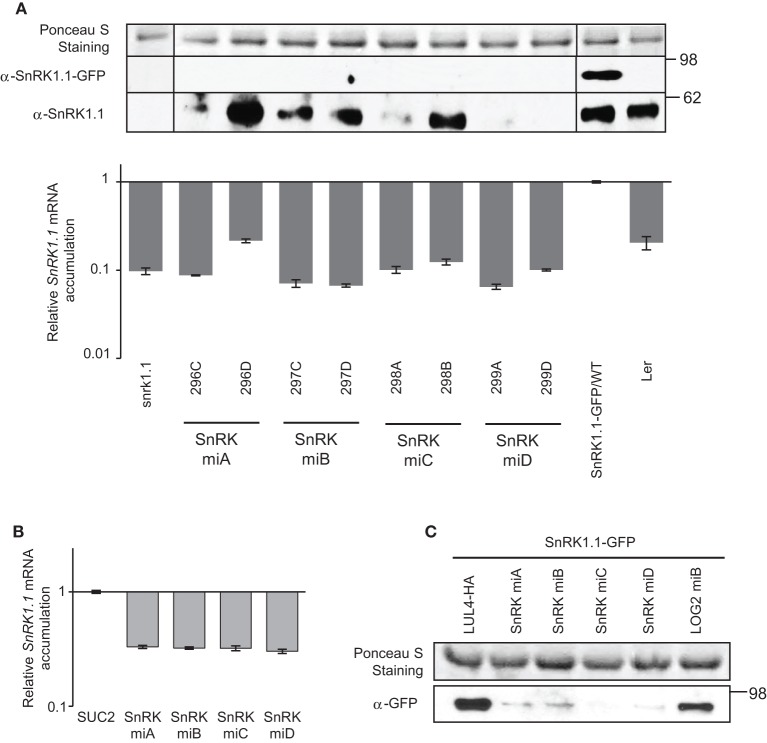
**Analysis of amiRNAs targeting *SnRK1.1* in stably transformed Arabidopsis and transiently transformed *N. benthamiana*. (A)** amiRNAs targeting *SnRK1.1* (SnRK miA, miB, miC, miD) were stably introduced into the SnRK1.1-GFP/WT(Ler) background. About 10 progenies of each line were analyzed. SnRK1.1 protein levels were determined by western blot using anti-SnRK1.1 antibodies. Endogenous SnRK1.1 and SnRK1.1-GFP blots are shown with different exposure time. *SnRK1.1* relative mRNA levels were measured by qRT-PCR (accumulation is expressed relative to the level of *SnRK1.1* in SnRK1.1-GFP/WT). Error bars represent standard error processed by qbase^PLUS^. **(B)** Relative *SnRK1.1* mRNA levels in transiently transformed *N. benthamiana*. *SnRK1.1* was co-expressed with *SUC2 or* amiRNA^SnRK1.1^. *SnRK1.1* mRNA level were estimated by qRT-PCR and expressed relative to levels in leaves transformed with *SnRK1.1* and *SUC2*. Errors bars represent standard error of at least two biological replicates. **(C)** Western blot showing effects of amiRNA^SnRK1.1^ on SnRK1.1 protein accumulation in transiently transformed *N. benthamiana*. amiRNA^LOG2^ (LOG2 miB) was used as a negative control, and co-expression of LUL4-HA (a paralog from LOG2; Pratelli et al., 2012) was used as co-expression control. Numbers on the right indicate molecular weight in kDa.

The four amiRNA^SnRK1.1^ were introduced into a 35S-SnRK1.1-GFP/WT (Landsberg) line, and transformants were selected similarly as the MIPS1 amiRNA lines above. The amiRNA^SnRK1.1^ reduced *SnRK1.1* transcripts abundance by 80–95% of SnRK1.1-GFP/WT levels, paralleled by a complete suppression of SnRK1.1-GFP protein accumulation (Figure 3A). Because the SnRK1.1-GFP construct was introduced into a wild type background and an anti-SnRK1.1 antibody was used, endogenous SnRK1.1 protein levels could be monitored. Contrary to the transgene, endogenous SnRK1.1 protein was reduced in four out of the eight lines studied, with 299A and 299D being the most potent, recapitulating the protein levels found in *snrk1.1* knock-down mutant (Figure 3A). It should be noted here that the suppression of the wild type SnRK1.1 protein accumulation could result from siRNAs generated from the degradation of the SnRK1.1-GFP mRNAs. In the transient assay in *N. benthamiana*, all amiRNA^SnRK1.1^ reduced *SnRK1.1* transcripts level equally, by 70%, less than in Arabidopsis, while SnRK1.1 protein accumulation was almost completely suppressed (Figures 3B,C). In conclusion, SnRK1.1 protein accumulation correlated with mRNA levels upon action of the four amiRNAs targeting *SnRK1.1* in *N. benthamiana*. In Arabidopsis, although the four amiRNA^SnRK1.1^ were equally sufficient to suppress SnRK1.1-GFP protein accumulation, amiRNA^SnRK1.1^-D was the most potent to suppress endogenous SnRK1.1 protein accumulation.

### Comparison of the effect of amiRNAs to suppress the expression of *GDU1*

**Figure 4:**
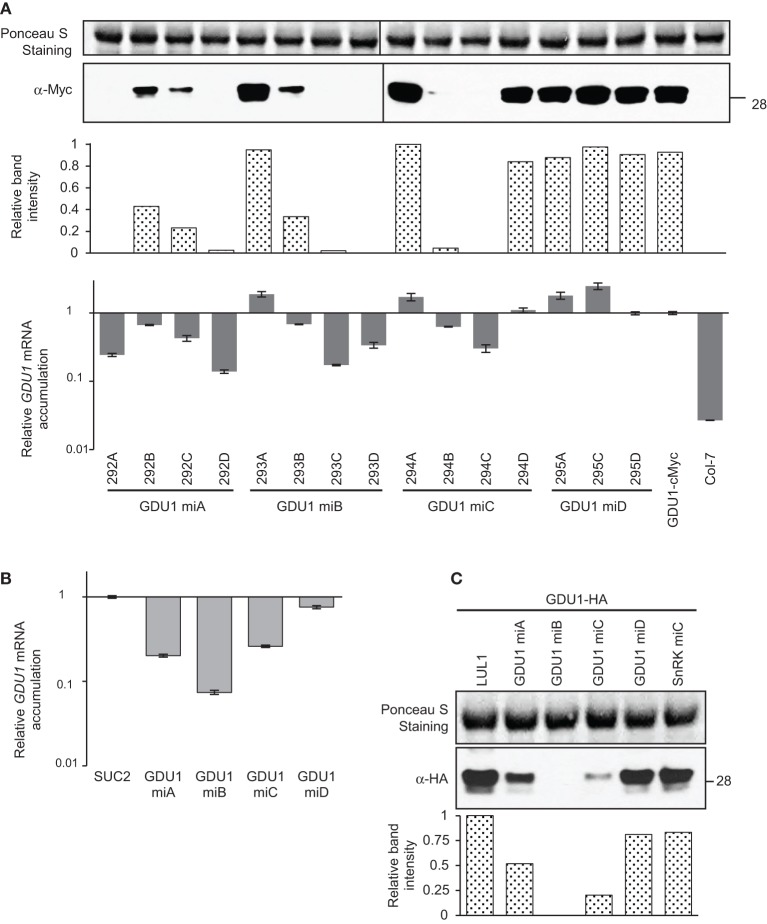
**Analysis of amiRNAs targeting *GDU1* in stably transformed Arabidopsis and transiently transformed *N. benthamiana*. (A)** amiRNAs targeting *GDU1* (GDU1 miA, miB, miC, and miD) were stably introduced into an Arabidopsis line expressing GDU1-cMyc under the control of the *GDU1* promoter. About four progenies of each transformant line were analyzed. GDU1 protein levels in stems were determined by western blot using an anti-cMyc antibody, band intensities are expressed relative to the maximum (line 294A). *GDU1* relative mRNA levels were measured by qRT-PCR (expressed relative to the *GDU1-cMyc* line). Error bars represent standard error processed by qbase^PLUS^. **(B)** Relative *GDU1* mRNA levels in transiently transformed *N. benthamiana*. *GDU1* was co-expressed with *SUC2* or amiRNA^GDU1^. *GDU1* mRNA level were estimated by qRT-PCR and expressed relative to levels in leaves transformed with *GDU1* and *SUC2*. Errors bars represent standard error of at least two biological replicates. **(C)** Western blot showing effects of amiRNA^GDU1^ on GDU1 protein accumulation in transiently transformed *N. benthamiana*, band intensities are expressed relative to the maximum (sample LUL1). amiRNA^SnRK1.1^ (SnRK miC) was used as a negative control, and co-expression of LUL1 (a paralog from LOG2; Pratelli et al., 2012) was used as co-expression control. Numbers on the right indicate molecular weight in kDa.

amiRNA^GDU1^ were introduced into a line expressing GDU1-cMyc under the control of its own promoter (Pilot et al., 2004). Since the parental line did not display any visible phenotype, no simple visualization of the efficiency of each amiRNA could be obtained in T1, contrary to *LOG2*, *MIPS1*, and *SnRK1.1* studies above. Thus, four independent transformants from each transformation were randomly selected and studied. Two of the four lines expressing amiRNA^GDU1^-A, -B, and -C showed >98% suppression of GDU1 protein accumulation while none of the lines expressing amiRNA^GDU1^-D did (Figure 4A). Surprisingly, lines accumulating GDU1 protein at the same level as the parental lines (lines 293A, 294A, 294D, 295A, and 295C) showed an apparent increase in *GDU1* mRNA accumulation, reminiscent of the effect of amiRNA^LOG2^-C and -D on *LOG2* mRNA in *N. benthamiana* (see Figure 1B). Reduction of GDU1-cMyc protein was always accompanied by reduction in *GDU1* mRNA: the strongest decrease in *GDU1* mRNA accumulation always corresponded to the strongest reduction in GDU1 protein content (Figure 4A). In the *N. benthamiana* transient assay, amiRNA^GDU1^-A, -B and -C reduced *GDU1* transcript levels by 80, 95, and 75%, respectively, but amiRNA^GDU1^-D had no effect. Similar to Arabidopsis, a strong correlation between *GDU1* mRNA and protein contents could be observed in this system (Figures 4B,C). The different silencing efficiency of the four amiRNA^GDU1^ was also observed by fluorescence microscopy when co-expressing amiRNA^GDU1^ with GDU1-GFP in *N. benthamiana* leaves (data not shown), and was in accordance with the above results.

### Testing the relationship between amiRNA, target mRNA and protein accumulations

These results suggested that the efficacy of amiRNA varies greatly from one amiRNA to another, and that even amiRNAs found poorly efficient in *N. benthamiana* are sometimes able to suppress the expression of the target genes when stably expressed in Arabidopsis.

**Figure 5:**
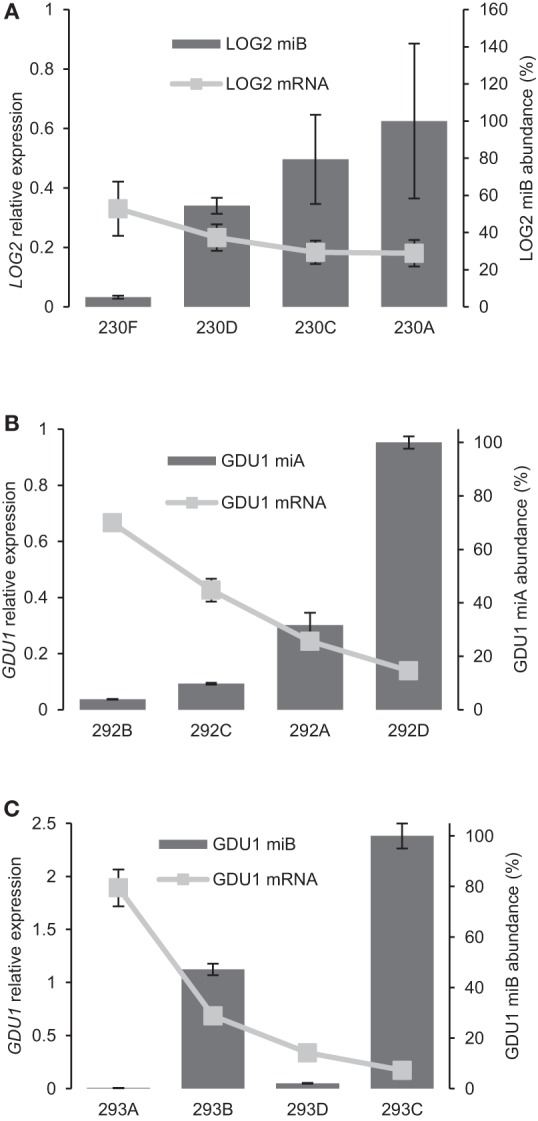
**Analysis of the relationship between abundance of amiRNA and target mRNAs. (A)** amiRNA^LOG2^-B abundance determined by qRT-PCR for the same lines as in Figure 1A. Accumulation is expressed relative to the highest expression (line 230A). *LOG2* mRNA level is expressed relative to levels in *gdu1-1D*. **(B,C)** amiRNA abundance determined by qRT-PCR for amiRNA^GDU1^-A **(B)** and amiRNA^GDU1^-B **(C)** for the same lines as in Figure 4A. Accumulations are expressed relative to the highest expression (lines 292D and 293C respectively). *GDU1* mRNA level is expressed relative to levels in the *GDU1-cMyc* line.

To test whether different expression of the amiRNAs in different Arabidopsis lines could explain this latter observation, amiRNA accumulation was determined by quantitative RT-PCR (Chen et al., 2005; Schmittgen et al., 2008). A slight decrease in *LOG2* mRNA accompanied increase in amiRNA^LOG2^-B abundance (Figure 5A). Nevertheless, the change in LOG2 mRNA was modest (from 65 to 80% reduction compared to the *gdu1-1D* parent), while the change in amiRNA^LOG2^-B expression varied over 20-folds. The phenotype of the plants correlated well with amiRNA^LOG2^-B accumulations but not with the reduction in *LOG2* mRNA level (compare Figure 1A and Figure 5A): phenotype suppression was absent in line 230F which expressed amiRNA^LOG2^-B ten times less than 230D, yet *LOG2* mRNA abundance was reduced by 65 and 75% from the *gdu1-1D* parent in these lines, respectively. For GDU1, a clear negative correlation was observed between the expression of both amiRNA^GDU1^-A and -B and *GDU1* mRNA accumulation (Figures 5B,C) in Arabidopsis. The only discrepancy is the low GDU1 mRNA accumulation in line 293D while amiRNA^GDU1^-B is not expressed at very high levels, which remains unexplained. The accumulation of amiRNA^GDU1^-A (2 to 50 times more than the ACT2 mRNA levels, as measured by q-RT-PCR) seemed higher than the accumulation of amiRNA^GDU1^-B (0.01 to 5 times the ACT2 mRNA levels). It should be noted that the amplification efficiency of each amiRNA could be different, and the above results might reflect this difference. As a comparison, the levels of the efficient amiRNA^LOG2^-B were estimated as 0.005 to 0.1 times the *ACT2* mRNA accumulation (data not shown). These data support the above observation that amiRNA^LOG2^-B and amiRNA^GDU1^-A and -B act differently on the stability of the target mRNA.

To test whether the difference in amiRNA^LOG2^-B abundance could cause differences in *LOG2* mRNA cleavage at the binding site, primers flanking amiRNA^LOG2^-B binding site were designed. *LOG2* mRNA levels were quantitated by the flanking primer pair and a non-flanking primer pair, and no difference was found between the two quantities in any of the four tested lines (Figure S1B). This result indicates that the cleaved mRNA does not accumulate, otherwise the non-flanking pair should have detected more *LOG2* mRNA than the flanking pair, suggesting that the cleavage products are quickly degraded. The *LOG2* mRNA species quantitated by the qPCR correspond thus to the full length, translatable, *LOG2* mRNA.

**Figure 6:**
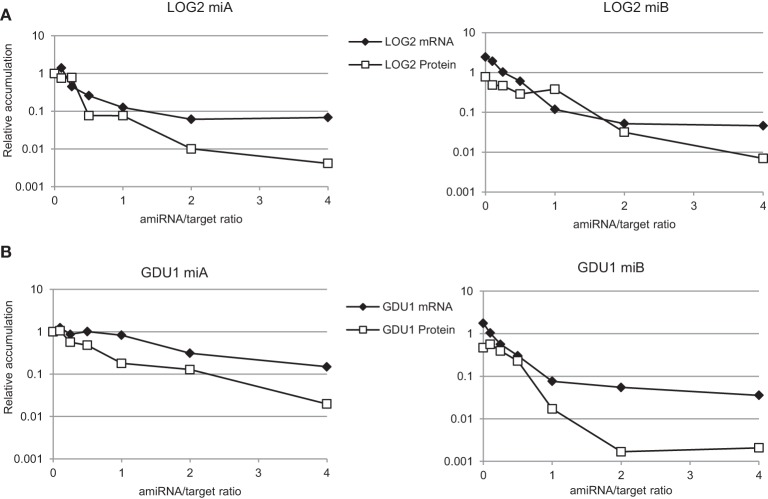
**Analysis of the relationship between amiRNA expression, target mRNA and protein accumulations**. Two amiRNAs targeting **(A)** *LOG2* and **(B)** *GDU1*. Leaves of *N. benthamiana* were infiltrated with various ratios of amiRNA / target gene (*mLOG2-HA* or *GDU1-HA*), ranging from 0.1 to 4. Control (ratio 0, left panels) corresponds to co-expression of the target gene with *SUC2*. The ratio 0 for the right panels corresponds to co-expression of the target genes with amiRNA^SnRK1.1^-C. The same amount of the target constructs was infiltrated for each sample. Variable amounts of amiRNA^GDU1^ (or amiRNA^LOG2^) and amiRNA^SnRK1.1^-C were co-infiltrated to keep constant the total amount of amiRNA expressed in the leaf. Target mRNA and protein levels were determined by qRT-PCR and western blot respectively. Error bars represent standard error processed by qbase^PLUS^. Levels are expressed relative to the 0 ratio of the left panels.

The effect of the intensity of the expression of various amiRNAs on protein accumulation was tested in *N. benthamiana* by varying the ratios of *Agrobacterium* strains delivering the various constructs in the plant cells. The amount of target construct being kept constant, the amount of amiRNA used varied from 1/10 to four times the amount of target construct. amiRNA^LOG2^-A and -B affected LOG2 mRNA and protein content in a very similar way, in good agreement with their similar efficacy noted earlier (Figure 6A). With increasing amiRNA^LOG2^/LOG2 ratio, the effect on mRNA leveled off to about 90% reduction, while protein content decreased down to 1% of the control (Figure 6A), suggesting an greater effect of the amiRNA on protein rather than mRNA content, especially at higher amiRNA expression. Similarly, amiRNA^GDU1^-A and -B affected more strongly protein than mRNA content (Figure 6B), but the efficacy of these amiRNAs was different. As noted previously, amiRNA^GDU1^-B could suppress more efficiently *GDU1* expression than amiRNA^GDU1^-A. While increasing amiRNA^GDU1^-A expression led to a parallel decrease in both *GDU1* mRNA and protein accumulation, increase in amiRNA^GDU1^-B expression led to leveling off of *GDU1* mRNA suppression while GDU1 protein content further decreased (Figure 6B). amiRNA^GDU1^-A and -B seem to have different modes of action, amiRNA^GDU1^-B possibly affecting more translation than amiRNA^GDU1^-A. This hypothesis could explain the complete suppression of GDU1 protein accumulation by amiRNA^GDU1^-B in *N. benthamiana* (Figure 4C), compared to amiRNA^GDU1^-A.

## Discussion

### Strong activity of amiRNAs at the translational level

The results of this study are summarized in Table S3. We found that in *N. benthamiana* leaves, a decrease in mRNA content parallels a decrease in protein content (for 15 out of 16 amiRNAs tested, the exception being amiRNA^MIPS1^-C). Nevertheless, the reduction of the target mRNA by amiRNA is most of the time modest (less than five times), while the decrease in target protein accumulation is much stronger (more than 10 times). For *MIPS1* and *SnRK1.1*, no linear relationship could be observed by comparing mRNA content and protein accumulation, and it rather appeared that protein accumulation was suppressed when a threshold in mRNA decrease was met (Figures 2, 3). The relationship was much more linear for *GDU1* (Figure 4). This suggests that measuring the silencing efficacy of amiRNAs based on the target mRNA level would not perfectly reflect their ability at suppressing the target protein accumulation or activity.

The discrepancy between the intensity of the decreases in target mRNA and protein accumulations observed for *LOG2*, *MIPS1*, and *SnRK1.1* suggests that the decrease in target mRNA level is not the only reason for decrease in target protein content. *N. benthamiana* assays for amiRNA^LOG2^-A and -B and amiRNA^GDU1^-B support this hypothesis (Figure 6). Reports by Li et al. for amiRNAs (Li et al., 2013a) and several recent articles for endogenous miRNAs (Aukerman and Sakai, 2003; Chen, 2004; Gandikota et al., 2007; Brodersen et al., 2008; Dugas and Bartel, 2008; Lanet et al., 2009; Zhu et al., 2009; Beauclair et al., 2010; Zhu and Helliwell, 2011; Alonso-Peral et al., 2012; Li et al., 2013b; Ma et al., 2013; Meijer et al., 2013) support the fact that miRNAs in plants can act at the translation level, in addition to initiating mRNA cleavage. The expression of the target gene is likely being repressed by a combination of mRNA cleavage and translational inhibition. Our results support this observation, and agree with previous results for amiRNAs (Li et al., 2013a), which demonstrated that assessing amiRNA efficiency at the level of the target protein accumulation is more appropriate than at the target mRNA level.

### Use of *N. benthamiana* to test amiRNA efficiency is valid

By transiently expressing amiRNAs with the corresponding target gene in Arabidopsis mesophyll protoplasts, Kim and Somers were able to rapidly find amiRNAs efficiently silencing known genes that recapitulates loss-of-function mutant phenotype of the circadian clock (Kim and Somers, 2010). In a more comprehensive assay, Li et al. used Arabidopsis protoplasts to transiently express a protein and the corresponding amiRNAs to be tested and developed epitope-tagged protein-based amiRNA (ETPamir) screens to facilitate validation of optimal amiRNAs (Li et al., 2013a). While well described and used, we found that preparation of protoplasts requires some experience, and might pose troubles in laboratories where this technique is not established. Transient gene expression in *N. benthamiana* leaves is often viewed as a simpler method, used by many groups. *N. benthamiana* has been used to test for amiRNA expression before stable transformation of potato and *N. benthamiana* (Bhagwat et al., 2013). The authors of this report found a trend between the strength of expression of amiRNA in the transient assay and stable transformation, suggesting that amiRNA could be selected for higher expression in *N. benthamiana* before stable transformation. Nevertheless, the efficacy at suppressing target gene expression, neither at the target mRNA level, nor at the target protein level was tested in this study (Bhagwat et al., 2013). More recently, transient expression in *N. benthamiana* has been used to study complementarity requirement of miRNAs for their target to efficiently induce suppression (Liu et al., 2014). The *N. benthamiana* system offers several advantages: (1) It directly assesses amiRNA silencing efficacy by measuring target protein accumulation, which circumvents the complexity of the mode of action of amiRNA at the target mRNA and/or the target protein level. Fusion of a tag, such as HA and cMyc, to target proteins enables measuring protein accumulation in plants, with minimal interference with target protein function. The use of GFP can facilitate selection of efficient amiRNAs at the seedling stage and provides direct visualization by microscopy, but may interfere with target protein function and stability. (2) Expressing target protein and amiRNA candidates at the same time is simple and fast. As long as amiRNA candidates and their tagged target protein constructs are prepared and introduced into *Agrobacterium*, transient expression of amiRNAs and their target in *N. benthamiana* requires minimum preparation. Two days after infiltration, the target mRNA and/or protein levels can be measured and the most efficient amiRNA can be determined. (3) Many amiRNA candidates can be tested in parallel, with various target construct/amiRNA ratios if necessary. (4) Inefficient amiRNAs (e.g., amiRNA^LOG2^-C and -D; amiRNA^GDU1^-D) can be discarded before considering stable plant transformation.

In addition to testing amiRNA efficiency in *N. benthamiana* by transient assay, we expressed all of the 16 amiRNAs in Arabidopsis and tested their effect on target protein levels and corresponding phenotypic changes to confirm the reliability of results from the transient assay. Indeed, amiRNAs that are more potent to suppress target protein accumulation in the transient assay were more likely to suppress target protein accumulation in stably transformed Arabidopsis and cause expected phenotypic changes (e.g., amiRNA^LOG2^-A and -B; amiRNA^GDU1^-B and -C). amiRNAs that suppressed target protein accumulation with equal efficiency in the transient assay exhibited similar equal efficiency to suppress target protein accumulation in stably transformed Arabidopsis (e.g., amiRNA^MIPS1^ and amiRNA^SnRK1.1^). Li et al. expressed only six of 63 amiRNAs in Arabidopsis and observed phenotypic change of four lines (another one was modest; Li et al., 2013a). Surprisingly, even our less efficient amiRNA in *N. benthamiana* (amiRNA^MIPS1^-C) was able to reduce target protein accumulation and cause the corresponding phenotypic changes in stably transformed Arabidopsis (Figure 2). On the other hand, amiRNAs found efficient in *N. benthamiana* did not always lead to strong decrease in protein accumulation in every transformed Arabidopsis line (see amiRNA^MIPS1^-A, -B, and -D, and amiRNA^GDU1^-B, Figures 2, 4). This observation can be attributed to the different expression level of the amiRNAs amongst different transformants, as observed for amiRNA^LOG2^-B, and amiRNA^GDU1^-A and -B (Figure 5). This indicates that expression levels of the amiRNA are important for its efficacy in stably transformed plants, and likely depend on number of T-DNA copies inserted in the genome and localization of the insertion(s). In conclusion, this assay will enable discarding non-efficient amiRNAs, but cannot determine the most efficient ones.

In the transient assay, the target proteins are driven by CaMV 35S promoter and epitope tagged to allow estimation of amiRNA silencing efficiency by measuring the target protein accumulation. However, in most cases, the real target of those amiRNAs is an endogenous gene. In this study, we have verified the efficiency of amiRNA to suppress target expression under various genetic backgrounds in Arabidopsis. amiRNAs targeting *LOG2* were introduced into *gdu1-1D* background. amiRNA^LOG2^-A and -B, which were shown potent to suppress *LOG2* expression in the transient assay, successfully reduced LOG2 activity and abolished the LOG2-dependent Gdu1D phenotype in Arabidopsis. amiRNA^GDU1^ were introduced into a line expressing GDU1-cMyc under GDU1 promoter, where *GDU1* mRNA accumulation is only slightly increased compared to the wild type (data not shown). amiRNA^GDU1^-D, which was found inefficient in the transient assay, could not suppress GDU1 protein accumulation in Arabidopsis. The four amiRNA^MIPS1^ showed similar modest efficiency to suppress MIPS1 protein accumulation in the transient assay. In the 35S-MIPS1-GFP/*mips1-2* background, one out of the two lines selected for each amiRNA^MIPS1^ showed reduced MIPS1 protein accumulation and the corresponding phenotypic changes. In summary, the silencing efficiency of amiRNAs measured by the transient assay in *N. benthamiana* represents well their efficiency in stably transformed Arabidopsis, no matter if the target is a transgene or an endogenous gene.

### Analysis of free energy and target site location of amiRNAs

Li et al. proposed a new amiRNA selection criteria, in which optimal amiRNAs should have no predicted off-target genes, complementary sequence within the first 5′ 200 nucleotides of the coding sequence, displaying up to two mismatches in position 1 or 15–21, and with an hybridization energy larger than 80% of the perfect match (Li et al., 2013a). The hybridization energy of our optimal amiRNAs varied from 78 to 99%, in good agreement with the published results. However, we found several amiRNAs not meeting these criteria that also efficiently silenced target gene expression, with no clear preference for the target site location on the target transcript (Table S2). Our results indicate that the mode of action of an efficient amiRNA involves many different factors, making necessary to test experimentally the amiRNAs to be selected for stable plant transformation. Similar to Li et al., we could not detect any mRNA cleavage product by using a quantitative RT-PCR approach, suggesting that the cleavage products are quickly degraded (Li et al., 2013a). The reduction of target mRNA level does not always correlate with decreases in target protein accumulation, and is thus an insufficient measure to determine optimal amiRNAs. As also concluded by Li et al., we found that measuring target protein level by transient co-expression of amiRNAs with the target in heterologous system (*N. benthamiana* leaves or in protoplasts) is a better way to find optimal amiRNAs (Li et al., 2013a).

## Materials and methods

### Plant lines and transformations

*Arabidopsis thaliana* and *N. benthamiana* were grown under 120 μE/m^2^/s, 22°C 16 h light/8 h dark, or 40 μE/m^2^/s (low light condition) when needed. Plant lines 35S-MIPS1-GFP in the *mips1-2* mutant (ecotype Columbia; Donahue et al., 2010), 35S-SnRK1.1-GFP in wild type (Landsberg *erecta*) and *mips1-2* were obtained from Dr. Glenda Gillaspy (Virginia Tech). *snrk1.1* knock-down line (Baena-Gonzalez et al., 2007) was obtained from Dr. Glenda Gillaspy with permission from Dr. Filip Rolland. *A. thaliana* were transformed by the floral dip method (Clough and Bent, 1998) using *Agrobacterium tumefaciens* GV3101 (pMP90). Phenotypes were recorded about a month after germination. For transient expression of proteins in *N. benthamiana*, young leaves of 5-week old plants were infiltrated with a suspension of *A. tumefaciens* carrying the constructs of interest according to Batoko et al. (2000), with the following modifications. The bacteria were grown overnight in LB supplemented with appropriate antibiotics, washed twice in 10 mM MgCl_2_, 100 μM acetosyringone, and diluted to final OD_600_ of 0.05 (target constructs) and 0.1 (amiRNA constructs) in the same solution before infiltration in *N. benthamiana* leaves.

### Cloning and constructs

Primer sequences used for cloning are listed in Table S4. Sequences of the amiRNAs were obtained from WMD (http://wmd3.weigelworld.org/; Schwab et al., 2006; Ossowski et al., 2008) following the guidelines of the website, and were created by single overlap extension PCR of three fragments as described previously (Pratelli et al., 2012). The primers corresponding to pRS300 (Schwab et al., 2010) used for amplification of the amiRNAs contained the Gateway attB sites. The final PCR fragment was cloned into pDONRZeo (Life Technologies), sequenced, and transferred into the pSWsNkan or pSWSNhyg binary vector, derivatives of pJHA212K (Yoo et al., 2005; Pratelli and Pilot, unpublished data), between the CsVMV promoter (Verdaguer et al., 1998) and the terminator of the small subunit of the Rubisco from pea (Pisum sativum; accession no. X00806). The MIPS1-GFP construct has been previously described (Donahue et al., 2010). The SnRK1.1-GFP construct was obtained from Dr. Gillaspy, Virginia Tech (unpublished). mLOG2-HA (whose ubiquitin ligase activity has been abolished by mutagenesis), GDU1-cMyc and GDU1-HA constructs were previously described (Pratelli et al., 2012).

### RNA extraction and quantitative RT-PCR

Total RNA were extracted from leaves with TRI Reagent^®^ (Sigma-Aldrich) following manufacturer's instructions. The integrity of the RNA was confirmed by agarose gel electrophoresis before reverse transcription. cDNAs were synthesized from 2 μg of total RNA using the SuperScript III (LifeTechnologies) according to the manufacturer's instructions, in a 10 μl reaction volume. Five μl of primer mix (1 μM each) and 5 μl of the reverse transcription products diluted 50 times in water were mixed with 10 μl of 2× SYBR^®^ Green PCR Master Mix (LifeTechnologies) and subjected to the following cycles: 2 min 50°C, 10 min 95°C, 40 times of 15 s 95°C, 15 s 55°C, 1 min 72°C in a 7300 Real Time PCR System, Applied Biosystems. Three reference genes (Actin2—AT3G18780, UBC9—AT4G27960, and PP2A—AT1G13320) were tested in the same experimental conditions. Results were processed with qbase^PLUS^ software (Biogazelle; Vandesompele et al., 2002; Hellemans et al., 2007). qRT-PCR results were normalized and processed by the reference gene(s) selected from qbase^PLUS^ (Hellemans et al., 2007). amiRNA analysis by qPCR was performed following a previously described method (Chen et al., 2005; Schmittgen et al., 2008).

### Protein extraction and western blot

Proteins were extracted from infiltrated *N. benthamiana* leaves by homogenization in extraction buffer in a mortar on ice, or from stably transformed *Arabidopsis* tissues, ground in liquid nitrogen. Samples (300 mg fresh weight) were mixed with 1 ml extraction buffer [50 mM Tris-HCl pH 7.3, 150 mM NaCl, 10 mM MgCl_2_, 0.5% Nonidet P-40, 10 mM DTT, 1× Complete^®^ (Roche Diagnostics)]. Homogenates were centrifuged at 10,000 g, at 4°C for 15 min. Protein in the resulting supernatants were quantitated using the Bradford assay (Thermo Scientific), and 10 μg were analyzed by SDS-PAGE (4–12% polyacrylamide NuPAGE^®^ MES gel, Life Technologies). Proteins were transferred on a nitrocellulose membrane (GE Healthcare), and detected using anti-cMyc (Clone A-14, Santa Cruz; 1:10,000), anti-HA (Clone 3F10, Roche Diagnostics; 1:5000), anti-GFP (Clone FL, Santa Cruz; 1:2000), or anti-SnRK1.1 (Dr. Gillaspy, Virginia Tech, unpublished; 1:1500) primary antibodies, anti-rabbit, anti-rat or anti-goat (1:2000–1:10,000; Thermo Scientific) secondary antibodies, and the ECL-Plus western blotting detection system (GE Healthcare), and recorded on X-ray films or CCD camera. Intensity of the bands on the film was measured after film scanning, and may not accurately reflect the dynamic range of the signal intensities.

### Fluorescence microscope imaging

GFP-labeled proteins expressed in *N. benthamiana* epidermis cells were visualized on a Zeiss HBO 100 microscope illuminating system on an Axio Imager.M1 microscope using an EC plan-NEOFLUAR 20× N.A. 0.5 objective (Carl Zeiss), with BP 515-565 emission filter. All images were captured with the same light intensity and the same configurations.

## Author contributions

Shi Yu and Guillaume Pilot designed the experiments, Shi Yu performed the experiments and Shi Yu and Guillaume Pilot wrote the paper.

### Conflict of interest statement

The authors declare that the research was conducted in the absence of any commercial or financial relationships that could be construed as a potential conflict of interest.
