# Supplementary material for: Testing the efficiency of plant artificial microRNAs by transient expression in Nicotiana benthamiana reveals additional action at the translational level
Source: Front Plant Sci. 2014 Nov 19;5:622. doi: 10.3389/fpls.2014.00622 (PMC4237044; doi:10.3389/fpls.2014.00622)
Supplement: Supplementary file 1 [file Table1.PDF]

Table S1: Summary of the detection tags used for each target gene and the corresponding Arabidopsis line number.

Target Gene	Fused Tag	Line No.	Background
MIPS1	GFP	278, 279, 280, 281	35S-MIPS1-GFP/*mips1-2*
LOG2	-	224, 226, 228, 230	*gdu1-1D/*(Col-7)
SnRK1.1	GFP	296, 297, 298, 299	35S-SnRK1.1-GFP/WT(L*er*)
GDU1	cMyc	292, 293, 294, 295	pGDU1-GDU1-cMyc/WT(Col-7)

Table S2. Properties of the amiRNAs used in this study.

AmiRNA	Target Gene	Hybridization Energy (kcal/mol)	Number of mismatches and location (bases)	Location of target from ATG / length of CDS (bp)	WMD Prediction	Meet selection criteria proposed by Li et al?	Phenotypic change or target protein reduction in progenies
amiRNA^{LOG2} A [a]	AT3G09770	-33.59 (78.33%)	3 (1, 14, 17)	838-858 / 1167	Favorable	No	Yes
	AT5G03200	-30.10 (70.20%)	4 (1, 4, 14, 17)	760-780 / 1014	Favorable	No	NT
amiRNA^{LOG2} B [a]	AT3G09770	-37.04 (80.77%)	2 (1, 18)	155-175 / 1167	Favorable	Yes	Yes
amiRNA^{LOG2} C [a]	AT3G09770	-29.06 (70.07%)	3 (1, 14, 18)	383-403 / 1167	Favorable	No	No
	AT5G03200	-29.06 (70.07%)	3 (1, 14, 18)	323-243 / 1014	Favorable	No	NT
amiRNA^{LOG2} D [a]	AT3G09770	-34.77 (94.00%)	2 (1, 21)	-146- -126 / 1167	Favorable	No	No
amiRNA^{MIPS1} A	AT4G39800	-35.09 (80.93%)	3 (1, 18, 21)	113-133 / 1536	Favorable	No	Yes
amiRNA^{MIPS1} B	AT4G39800	-36.03 (84.92%)	2 (1, 18)	471-491 / 1536	Favorable	No	Yes
amiRNA^{MIPS1} C	AT4G39800	-40.53 (96.36%)	3 (1, 16, 21)	1100-1120 / 1536	Favorable	No	Yes
amiRNA^{MIPS1} D	AT4G39800	-37.79 (86.81%)	2 (1, 18)	824-844 / 1536	Favorable	No	Yes
amiRNA$^{SnRK1.1}$ A	AT3G01090	-37.47 (81.03%)	2 (1, 18)	364-384 / 1608	Favorable	No	Yes
amiRNA$^{SnRK1.1}$ B	AT3G01090	-40.57 (98.85%)	1 (1)	1040-1060 / 1608	Favorable	No	Yes
amiRNA$^{SnRK1.1}$ C	AT3G01090	-36.94 (82.71%)	2 (1, 18)	1454-1474 / 1608	Favorable	No	Yes
amiRNA$^{SnRK1.1}$ D	AT3G01090	-39.28 (88.75%)	2 (14, 21)	672-692 / 1608	Favorable	No	Yes
amiRNA^{GDU1} A	AT4G31730	-34.69 (81.32%)	1 (18)	12-32 / 477	Favorable	Yes	Yes
amiRNA^{GDU1} B	AT4G31730	-39.68 (93.65%)	2 (1, 20)	450-470 / 477	Favorable	No	Yes
amiRNA^{GDU1} C	AT4G31730	-38.34 (86.84%)	2 (14, 16)	122-142 / 477	Favorable	Yes	Yes
amiRNA^{GDU1} D	AT4G31730	-37.95 (77.01%)	2 (14, 17)	307-327 / 477	Favorable	No	No

[a] described in Pratelli *et al.* (2012). "NT", not tested.

Table S3: Summary of amiRNAs effect in transiently transformed *N. benthamiana* and stably transformed Arabidopsis.

Target Gene	amiRNA	Transient expression in *N. benthamiana*		Stable transformation into Arabidopsis		
		Decrease in mRNA content [a]	Decrease in protein content [b]	Transformation No.	Decrease in mRNA content [c]	Decrease in protein content [d]
LOG2	miA	-	+	228	0/4	NT
	miB	-	+	230	0/4	NT
	miC	-	-	224	0/3	NT
	miD	-	-	226	0/1	NT
GDU1	miA	-	±	292	0/4	2/4
	miB	+	+	293	0/4	2/4
	miC	-	+	294	0/4	2/4
	miD	-	-	295	0/3	0/3
SnRK1.1	miA	-	+	296	1/2	2/2
	miB	-	+	297	2/2	2/2
	miC	-	+	298	0/2	2/2
	miD	-	+	299	1/2	2/2
MIPS1	miA	-	±	278	0/1	0/1
	miB	-	±	279	1/2	1/2
	miC	-	±	280	1/2	1/2
	miD	-	±	281	1/2	1/2

[a] "-", target mRNA was reduced by less than 90% compared to control (minor effects); "+", target mRNA accumulation was reduced by more than 90% compared to control (major effects).

[b] "-", negligible decrease in protein accumulation; "+", strong decrease in protein accumulation, "±", moderate decrease in protein accumulation.

[c] Fractions represent the number of lines showing less than 10% target mRNA accumulation compared to the control; the numerators represent the number of lines tested.

[d] Fractions represent the number of lines showing strong decrease in protein accumulation, the numerators represent the number of lines tested. "NT", not tested.

Table S4. Oligonucleotides used in this study.

Name	Sequence	Purpose
GDU1 miRA f	GATCAAACTTCGATTGTACCCTCCTCTCTTTTGTATTCCA	$amiRNA^{GDU1}$ A
GDU1 miRA r	AGGAGGGTACAATCGAAGTTTGATCAAAGAGAATCAATGA	$amiRNA^{GDU1}$ A
GDU1 miRA* f	AGGAAGGTACAATCGTAGTTTGTTCACAGGTCGTGATATG	$amiRNA^{GDU1}$ A
GDU1 miRA* r	GAACAAACTACGATTGTACCTTCCTACATATATATTCCTA	$amiRNA^{GDU1}$ A
GDU1 miRB f	GATTTGTAGTAGTTGTCTCGCAGCTCTCTTTTGTATTCCA	$amiRNA^{GDU1}$ B
GDU1 miRB r	AGCTGCGAGACAACTACTACAAATCAAAGAGAATCAATGA	$amiRNA^{GDU1}$ B
GDU1 miRB* f	AGCTACGAGACAACTTCTACAATTCACAGGTCGTGATATG	$amiRNA^{GDU1}$ B
GDU1 miRB* r	GAATTGTAGAAGTTGTCTCGTAGCTACATATATATTCCTA	$amiRNA^{GDU1}$ B
GDU1 miRC f	GATTAGTCCTAGCATAGTCGCTACTCTCTTTTGTATTCCA	$amiRNA^{GDU1}$ C
GDU1 miRC r	AGTAGCGACTATGCTAGGACTAATCAAAGAGAATCAATGA	$amiRNA^{GDU1}$ C
GDU1 miRC* f	AGTAACGACTATGCTTGGACTATTCACAGGTCGTGATATG	$amiRNA^{GDU1}$ C
GDU1 miRC* r	GAATAGTCCAAGCATAGTCGTTACTACATATATATTCCTA	$amiRNA^{GDU1}$ C
GDU1 miRD f	GATGTCGCCAGATACGTCCGCAACTCTCTTTTGTATTCCA	$amiRNA^{GDU1}$ D
GDU1 miRD r	AGTTGCGGACGTATCTGGCGACATCAAAGAGAATCAATGA	$amiRNA^{GDU1}$ D
GDU1 miRD* f	AGTTACGGACGTATCAGGCGACTTCACAGGTCGTGATATG	$amiRNA^{GDU1}$ D
GDU1 miRD* r	GAAGTCGCCTGATACGTCCGTAACTACATATATATTCCTA	$amiRNA^{GDU1}$ D
MIPS1 miRA f	GATAATCCATTGGTAAGTGGCACCTCTCTTTTGTATTCCA	$amiRNA^{MIPS1}$ A
MIPS1 miRA r	AGGTGCCACTTACCAATGGATTATCAAAGAGAATCAATGA	$amiRNA^{MIPS1}$ A
MIPS1 miRA* f	AGGTACCACTTACCATTGGATTTTCACAGGTCGTGATATG	$amiRNA^{MIPS1}$ A
MIPS1 miRA* r	GAAAATCCAATGGTAAGTGGTACCTACATATATATTCCTA	$amiRNA^{MIPS1}$ A
MIPS1 miRB f	GATAGTCGATATCAAGAACGCTGCTCTCTTTTGTATTCCA	$amiRNA^{MIPS1}$ B

MIPS1 miRB r	AGCAGCGTTCTTGATATCGACTATCAAAGAGAATCAATGA	amiRNA^{MIPS1} B
MIPS1 miRB* f	AGCAACGTTCTTGATTTCGACTTTCACAGGTCGTGATATG	amiRNA^{MIPS1} B
MIPS1 miRB* r	GAAAGTCGAAATCAAGAACGTTGCTACATATATATTCCTA	amiRNA^{MIPS1} B
MIPS1 miRC f	GATGATACCATTGCTAGTAACCTCTCTCTTTTGTATTCCA	amiRNA^{MIPS1} C
MIPS1 miRC r	AGAGGTTACTAGCAATGGTATCATCAAAGAGAATCAATGA	amiRNA^{MIPS1} C
MIPS1 miRC* f	AGAGATTACTAGCAAAGGTATCTTCACAGGTCGTGATATG	amiRNA^{MIPS1} C
MIPS1 miRC* r	GAAGATACCTTTGCTAGTAATCTCTACATATATATTCCTA	amiRNA^{MIPS1} C
MIPS1 miRD f	GATATTGATGAACGGGATTGCCTCTCTCTTTTGTATTCCA	amiRNA^{MIPS1} D
MIPS1 miRD r	AGAGGCAATCCCGTTCATCAATATCAAAGAGAATCAATGA	amiRNA^{MIPS1} D
MIPS1 miRD* f	AGAGACAATCCCGTTGATCAATTTCACAGGTCGTGATATG	amiRNA^{MIPS1} D
MIPS1 miRD* r	GAAATTGATCAACGGGATTGTCTCTACATATATATTCCTA	amiRNA^{MIPS1} D
LOG2 miRA f	GATTTAACCCATAGTGTCCGCTTTCTCTCTTTTGTATTCC	amiRNA^{LOG2} A
LOG2 miRA r	GAAAGCGGACACTATGGGTTAAATCAAAGAGAATCAATGA	amiRNA^{LOG2} A
LOG2 miRA* f	GAAAACGGACACTATCGGTTAATTCACAGGTCGTGATATG	amiRNA^{LOG2} A
LOG2 miRA* r	GAATTAACCGATAGTGTCCGTTTTCTACATATATATTCCT	amiRNA^{LOG2} A
LOG2 miRB f	GATATTAGGATAGGGAGTACCGGTCTCTCTTTTGTATTCC	amiRNA^{LOG2} B
LOG2 miRB r	GACCGGTACTCCCTATCCTAATATCAAAGAGAATCAATGA	amiRNA^{LOG2} B
LOG2 miRB* f	GACCAGTACTCCCTAACCTAATTTCACAGGTCGTGATATG	amiRNA^{LOG2} B
LOG2 miRB* r	GAAATTAGGTTAGGGAGTACTGGTCTACATATATATTCCT	amiRNA^{LOG2} B
LOG2 miRC f	GATGTTACGAATCGTTACGCCTTTCTCTCTTTTGTATTCC	amiRNA^{LOG2} C
LOG2 miRC r	GAAAGGCGTAACGATTCGTAACATCAAAGAGAATCAATGA	amiRNA^{LOG2} C
LOG2 miRC* f	GAAAAGCGTAACGATACGTAACTTCACAGGTCGTGATATG	amiRNA^{LOG2} C
LOG2 miRC* r	GAAGTTACGTATCGTTACGCTTTTCTACATATATATTCCT	amiRNA^{LOG2} C
LOG2 miRD f	GATTAAGGAATTACGAAAAGCAGTCTCTCTTTTGTATTCC	amiRNA^{LOG2} D

LOG2 miRD r	GACTGCTTTTCGTAATTCCTTAATCAAAGAGAATCAATGA	amiRNA^{LOG2} D
LOG2 miRD* f	GACTACTTTTCGTAAATCCTTATTCACAGGTCGTGATATG	amiRNA^{LOG2} D
LOG2 miRD* r	GAATAAGGATTTACGAAAAGTAGTCTACATATATATTCCT	amiRNA^{LOG2} D
SnRK1.1 miRA f	GATAATAGCTCACCAGAGTGCACCTCTCTTTTGTATTCCA	amiRNA$^{SnRK1.1}$ A
SnRK1.1 miRA r	AGGTGCACTCTGGTGAGCTATTATCAAAGAGAATCAATGA	amiRNA$^{SnRK1.1}$ A
SnRK1.1 miRA* f	AGGTACACTCTGGTGTGCTATTTTCACAGGTCGTGATATG	amiRNA$^{SnRK1.1}$ A
SnRK1.1 miRA* r	GAAAATAGCACACCAGAGTGTACCTACATATATATTCCTA	amiRNA$^{SnRK1.1}$ A
SnRK1.1 miRB f	GATTATCAGATAGTACGTCACAGCTCTCTTTTGTATTCCA	amiRNA$^{SnRK1.1}$ B
SnRK1.1 miRB r	AGCTGTGACGTACTATCTGATAATCAAAGAGAATCAATGA	amiRNA$^{SnRK1.1}$ B
SnRK1.1 miRB* f	AGCTATGACGTACTAACTGATATTCACAGGTCGTGATATG	amiRNA$^{SnRK1.1}$ B
SnRK1.1 miRB* r	GAATATCAGTTAGTACGTCATAGCTACATATATATTCCTA	amiRNA$^{SnRK1.1}$ B
SnRK1.1 miRC f	GATATTGGGCGACTTAACACCTGCTCTCTTTTGTATTCCA	amiRNA$^{SnRK1.1}$ C
SnRK1.1 miRC r	AGCAGGTGTTAAGTCGCCCAATATCAAAGAGAATCAATGA	amiRNA$^{SnRK1.1}$ C
SnRK1.1 miRC* f	AGCAAGTGTTAAGTCCCCCAATTTCACAGGTCGTGATATG	amiRNA$^{SnRK1.1}$ C
SnRK1.1 miRC* r	GAAATTGGGGGACTTAACACTTGCTACATATATATTCCTA	amiRNA$^{SnRK1.1}$ C
SnRK1.1 miRD f	GATAGAGTATCACACGACAGCTACTCTCTTTTGTATTCCA	amiRNA$^{SnRK1.1}$ D
SnRK1.1 miRD r	AGTAGCTGTCGTGTGATACTCTATCAAAGAGAATCAATGA	amiRNA$^{SnRK1.1}$ D
SnRK1.1 miRD* f	AGTAACTGTCGTGTGTTACTCTTTCACAGGTCGTGATATG	amiRNA$^{SnRK1.1}$ D
SnRK1.1 miRD* r	GAAAGAGTAACACACGACAGTTACTACATATATATTCCTA	amiRNA$^{SnRK1.1}$ D
PRS300 attB1 b	GACAAGTTTGTACAAAAAAGCAGGCTCAAGCTTGATATCGAATTCCTGC	Cloning amiRNAs in pDONR Zeo
PRS300 attB1 c	GACAAGTTTGTACAAAAAAGCAGGCTCAGTTGTAAAACGACGGCCAGT	Cloning amiRNAs in pDONR Zeo
Act2 Q f	GGTAACATTGTGCTCAGTGGTGG	qPCR Reference gene
Act2 Q r	AACGACCTTAATCTTCATGCTGC	qPCR Reference gene
PP2A Q f	GCGGTTGTGGAGAACATGATAC	qPCR Reference gene

PP2A Q r	CACAATTCGTTGCTGTCTTCTTTA	qPCR Reference gene
UBC9 Q f	CACAATTTCCAAGGTGCTGCTAT	qPCR Reference gene
UBC9 Q r	GGACAGTATTTGTGTCAGCCCAT	qPCR Reference gene
MIPS1 Q f	CCAGTTCAAATCCGAGGGAGA	MIPS1 qPCR
MIPS1 Q r	GCTGCTTCGACAGTGCGTTA	MIPS1 qPCR
GDU1 Af	ATGGCCGGAGAAGATTTGC	GDU1 qPCR
GDU1 Ar	CGCCTTCTCTCATCTTCTCTTCC	GDU1 qPCR
LOG2 +800 f	TTCGAGAAAGGACTTGGTCAGA	LOG2 qPCR
LOG2 +900 r	CTGCCTTAACCGCTAATGGATA	LOG2 qPCR
SnRK1.1 Q f	CAATGTTGTCAAGTTTGAAATTCAG	SnRK1.1 qPCR
SnRK1.1 Q r	GGACTCGGAGCTGAGCAAGA	SnRK1.1 qPCR
LOG2-clv Q f	CTCCTTCTTCTTCTCTTCCTCCA	LOG2 qPCR
LOG2-clv Q r	GGGAGGATGGTGGTAGTAAGAAG	LOG2 qPCR
miRNA^{LOG2} B RT	GTCGTATCCAGTGCAGGGTCCGAGGTATTCGCACTGGATACGACCCGGTA	RT of amiRNA^{LOG2} B
miRNA^{GDU1} A RT	GTCGTATCCAGTGCAGGGTCCGAGGTATTCGCACTGGATACGACGAGGGT	RT of amiRNA^{GDU1} A
miRNA^{GDU1} B RT	GTCGTATCCAGTGCAGGGTCCGAGGTATTCGCACTGGATACGACCTGCGA	RT of amiRNA^{GDU1} B
miRNA^{LOG2} B Q f	GCGGCGGTATTAGGATAGGGAG	amiRNA^{LOG2} B qPCR
miRNA^{GDU1} A Q f	GCGGCGGTCAAACTTCGATTGT	amiRNA^{GDU1} A qPCR
miRNA^{GDU1} B Q f	GCGGCGGTTTGTAGTAGTTGTC	amiRNA^{GDU1} B qPCR
miRNA U Q r	ATCCAGTGCAGGGTCCGAG	amiRNA qPCR
