# Supplementary material for: Testing the efficiency of plant artificial microRNAs by transient expression in Nicotiana benthamiana reveals additional action at the translational level
Source: Front Plant Sci. 2014 Nov 19;5:622. doi: 10.3389/fpls.2014.00622 (PMC4237044; doi:10.3389/fpls.2014.00622)
Supplement: Supplementary file 2 [file Image1.PDF]

A

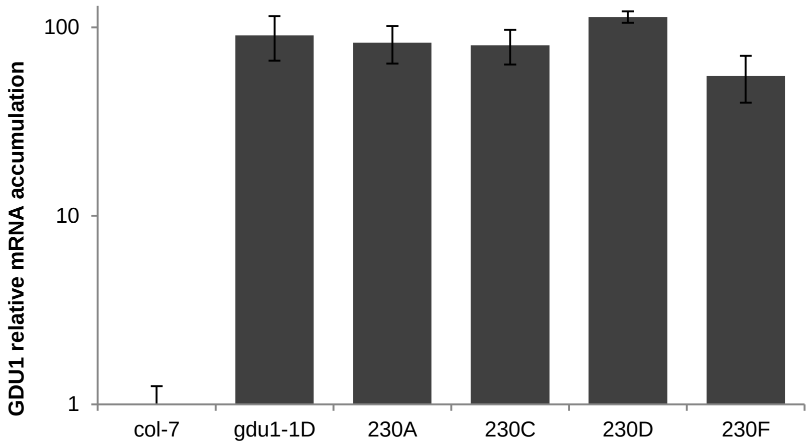

B

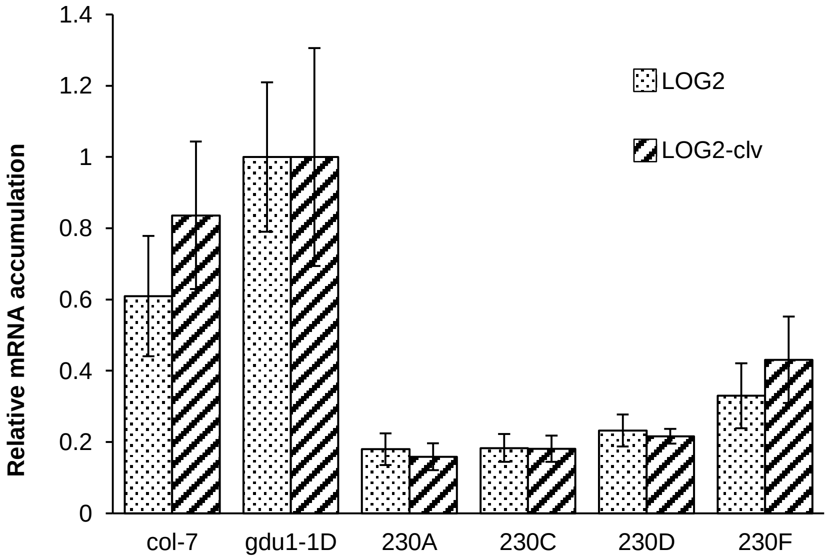

Supplemental Figure S1: **(A)** Accumulation of the *GDU1* mRNA in *gdu1-1D* lines expressing amiRNA^{LOG2} B measured by qRT-PCR. *GDU1* mRNA level is expressed relative to levels in Col7. **(B)** Accumulation of the *LOG2* mRNAs in *gdu1-1D* lines expressing amiRNA^{LOG2} B. The cleavage intensity of the LOG2 mRNA was tested by qRT-PCR using primers that flank the putative cleavage site (*LOG2-clv*). *LOG2* mRNA levels are expressed relative to levels in *gdu1-1D.*

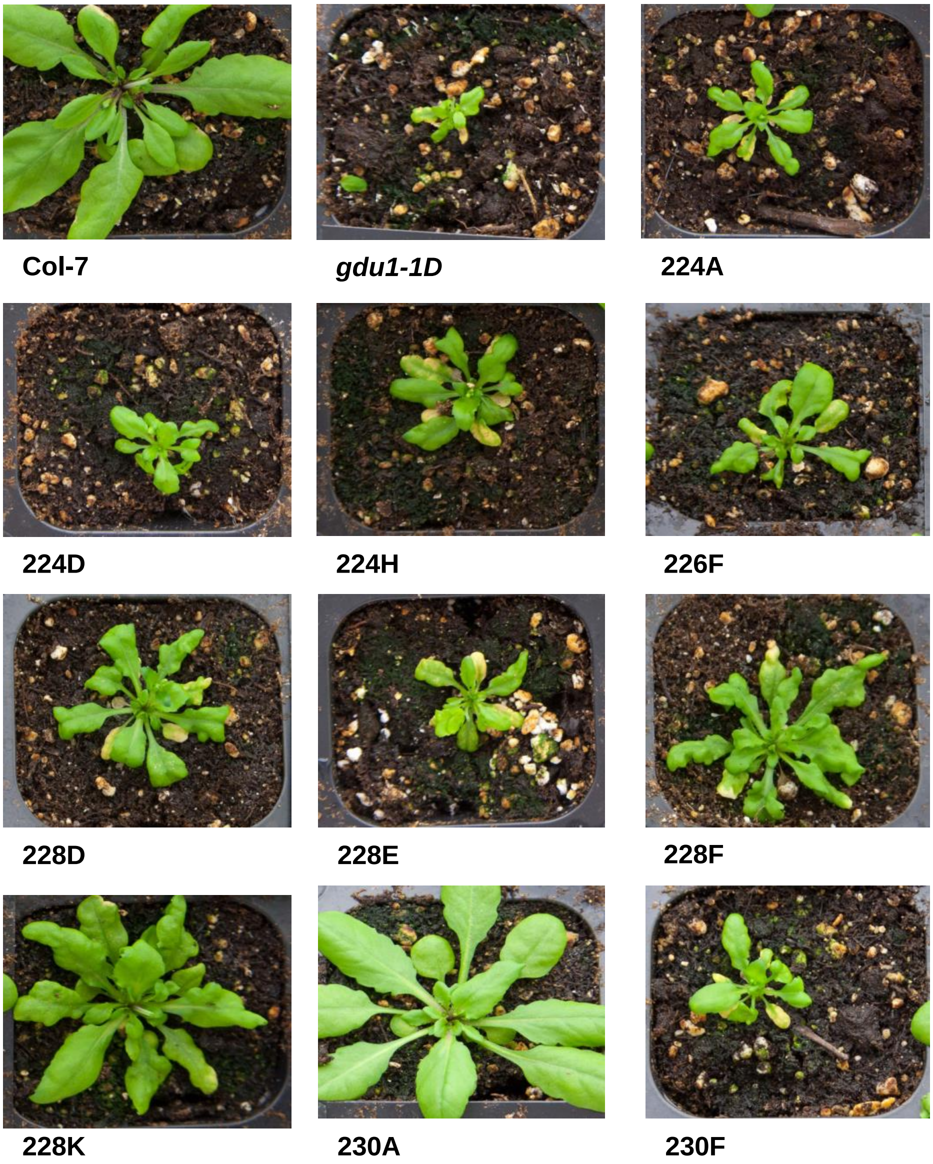

Supplemental Figure S2: Phenotype of wild type (Col-7), *gdu1-1D* and *gdu1-1D* plants expressing amiRNA^{LOG2}. Line numbers are same as Figure 1A.

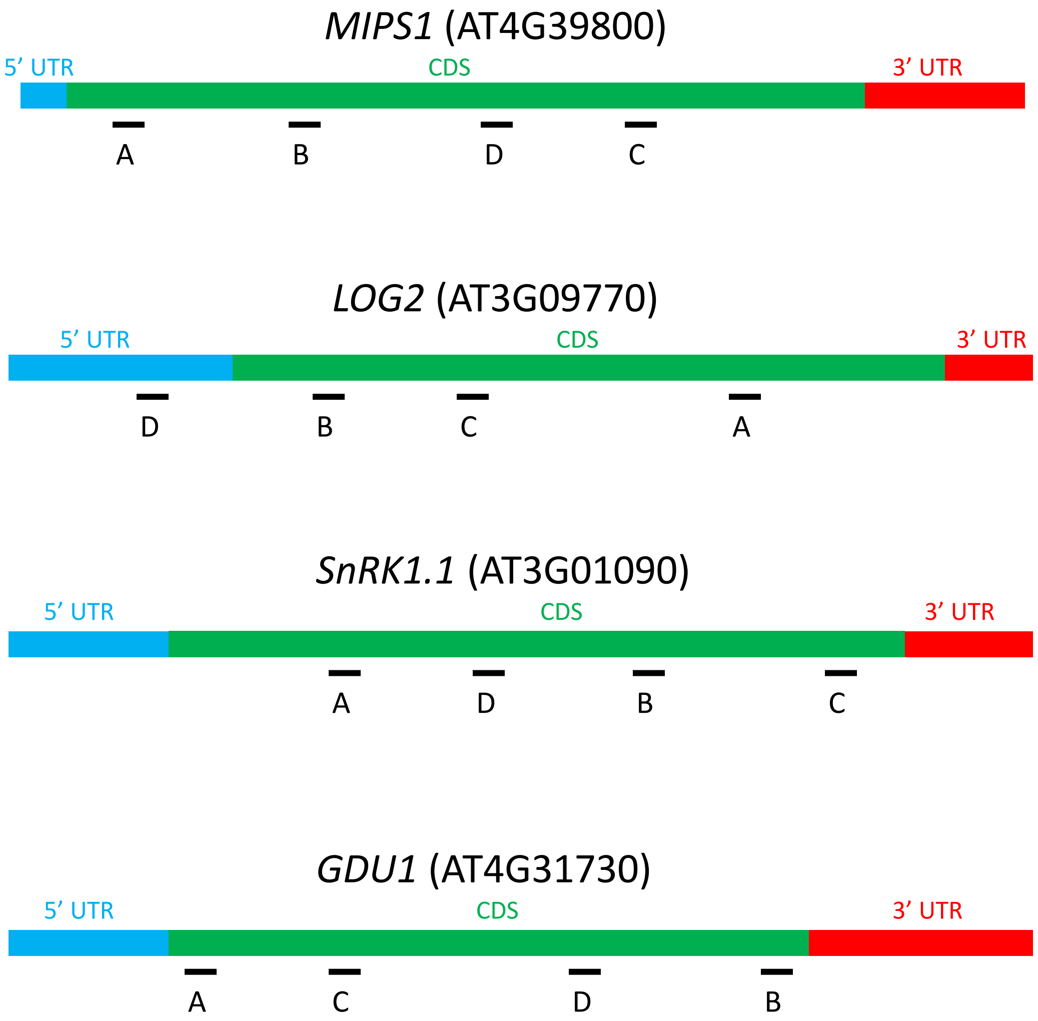

Supplemental Figure S3: Schematic figures showing the targeted location of each amiRNA (genes are not drawn to scale).

A

WMD3 - Web MicroRNA Designer

Transcript library:	TAIR8_cdna_20080412
Target genes:	AT5G03200.1,AT3G09770.1
Description:	R1-R2
Min. number of included targets:	2
Accepted off-targets:	0
Annotated:	1

Download xls

Targets: AT3G09770, AT5G03200

C	TGTTACGAATCGTTACGCCTT	-41.47	AT3G09770	-29.06	AT5G03200	-29.06
A	TTTAACCCATAGTGTCCGCTT	-42.88	AT3G09770	-33.59	AT5G03200	-30.10
	TATCGTTACGAATCGTGCCGA	-43.95	AT3G09770	-32.83	AT5G03200	-34.76
	TATCGTTACGAATCGTGACGA	-41.75	AT3G09770	-38.67	AT5G03200	-40.60
	TGTTACGAATCGTGATGCCTT	-42.61	AT3G09770	-33.93	AT5G03200	-33.93

B

WMD3 - Web MicroRNA Designer

Transcript library:	TAIR8_cdna_20080412
Target genes:	AT3G09770.1
Description:	R2
Min. number of included targets:	1
Accepted off-targets:	0
Annotated:	1

Download xls

D	TTAAGGAATTACGAAAAGCAG	-36.99	AT3G09770	-34.77
B	TATTAGGATAGGGAGTACCGG	-45.86	AT3G09770	-37.04
	TAATGACACTAATCCTTGCGG	-42.62	AT3G09770	-35.85
	TTGTTATTGGCGTTAAGCCCA	-42.55	AT3G09770	-39.52
	TTACTCGGCCGTGTTTACCTA	-44.74	AT3G09770	-37.02
	TGATATGATGAATGCGGTCAG	-43.53	AT3G09770	-36.38
	TAAATGACACTAATCCGTCCG	-42.00	AT3G09770	-34.23

C

Designer

Transcript library:	TAIR9_cdna_20090619
Target genes:	AT4G39800.1
Description:	MIPS1
Min. number of included targets:	1
Accepted off-targets:	0
Annotated:	1

Download xls

	TATACAAGTTACTTGACACCC	-41.03	AT4G39800	-34.75
	TATACAAGTTACTTCAGACCC	-41.27	AT4G39800	-34.99
	TAAAATATACCAACCCGTCCG	-41.60	AT4G39800	-39.22
	TATAACGCTCTGTGCTAGCTG	-45.29	AT4G39800	-37.05
	TAAAATATACCAACCCGTCCT	-40.80	AT4G39800	-38.18
	TCTTTGTTCGCAATCACGCGA	-43.83	AT4G39800	-38.77
	TTTACTATCCGCAACCTACGG	-44.10	AT4G39800	-36.86
	TATACAAGTTACTTCACACCC	-41.03	AT4G39800	-39.69
	TTTCACCGTTAAAAGATGCGA	-39.51	AT4G39800	-34.11
	TTGACCCGAAGTAATTGGCAT	-43.01	AT4G39800	-36.11
	TTAACGCTCTGTGTTAGGCGC	-46.31	AT4G39800	-37.51
	TAGACCAAACTAAAAGCGCTT	-39.97	AT4G39800	-31.97
A	TAATCCATTGGTAAGTGGCAC	-43.36	AT4G39800	-35.09
B	TAGTCGATATCAAGAACGCTG	-42.43	AT4G39800	-36.03
C	TGATACCATTGCTAGTAACCT	-42.06	AT4G39800	-40.53
	TAAGTCGATATCAAGAGCCGT	-43.21	AT4G39800	-37.51
	TAATCCATTGGTAAATGCCAA	-38.99	AT4G39800	-34.52
D	TATTGATGAACGGGATTGCCT	-43.53	AT4G39800	-37.79

D

Designer

Transcript library:	TAIR9_cdna_20090619
Target genes:	AT3G01090.1
Description:	SnRK1.1
Min. number of included targets:	1
Accepted off-targets:	0
Annotated:	1

Download xls

A	TAATAGCTCACCAGAGTGCAC	-46.24	AT3G01090	-37.47
	TAACTTGCCCGAAATTACCGC	-43.64	AT3G01090	-39.23
B	TTATCAGATAGTACGTCACAG	-41.04	AT3G01090	-40.57
	TTTAGTATTCAGAGGACGCGG	-44.33	AT3G01090	-37.74
	TAACTTGCCCGAAATTACCTG	-41.90	AT3G01090	-40.21
	TTATTTGAGTAGAGCACGCAT	-41.03	AT3G01090	-40.56
	TAGTATCAGATAGTACGGCAC	-43.77	AT3G01090	-35.42
	TAGTATCAGATAGTATGTCAC	-40.13	AT3G01090	-39.19
	TATAGTACGTCACAGTGCCAC	-45.56	AT3G01090	-41.85
C	TATTGGGCGACTTAACACCTG	-44.66	AT3G01090	-36.94
	TAATAAGTGGGGATTATTCTG	-38.82	AT3G01090	-35.58
	TTATTTGAGTAGAGGACGCAT	-41.25	AT3G01090	-35.13
	TATGTTGTAGTGCCGTATCTT	-41.16	AT3G01090	-36.42
	TCTCTATTATGCTGGACGCGT	-45.58	AT3G01090	-38.99
D	TAGAGTATCACACGACAGCTA	-44.26	AT3G01090	-39.28
	TGTTTAGTATTCAGAGGACTT	-39.37	AT3G01090	-38.94

E

Designer

Transcript library: TAIR9_cdna_20090619

Target genes: AT4G31730.1

Description: GDU1

Min. number of included targets: 1

Accepted off-targets: 0

Annotated: 1

Download xls

	TATTTCTAGGAATCATGACGT	-39.16	AT4G31730	-37.62
A	TCAAACTTCGATTGTACCCTC	-42.66	AT4G31730	-34.69
	TATTGTACGCTCAATGGTCAC	-42.93	AT4G31730	-38.72
	TCAAACTTCGATTGGACGCTC	-44.25	AT4G31730	-36.60
B	TTTGTAGTAGTTGTCTCGCAG	-42.37	AT4G31730	-39.68
C	TTAGTCCTAGCATAGTCGCTA	-44.15	AT4G31730	-38.34
	TTGATTGCGGCGTGATTCCGT	-46.35	AT4G31730	-40.75
	TATTTCTAGGAATAATGACGA	-36.82	AT4G31730	-32.15
	TAACTTCGATTGTACGCCCAA	-42.28	AT4G31730	-33.16
	TTGATTAACGGAGGTTGACCT	-43.09	AT4G31730	-38.09
	TAAGGCGATTAGTCCTAGCGT	-45.28	AT4G31730	-41.87
	TAAGGCGATTAGTTCTAGCAT	-41.25	AT4G31730	-39.51
	TTGATTGCGGCGTAATTCCGT	-43.77	AT4G31730	-38.75
	TAACTTCGATTGTACGGTCAA	-40.08	AT4G31730	-33.33
	TTGTACGCTCAATTGTCTCAC	-42.64	AT4G31730	-35.66
	TTGATTAACGGAGGTTCACGT	-42.25	AT4G31730	-34.84
	TATTGTACGCTCAATGGTCTA	-41.40	AT4G31730	-39.68
	TTACCCTCGTGACTACCTCAC	-47.91	AT4G31730	-42.64
	TATTTCTAGGAATTATGACGA	-36.82	AT4G31730	-36.85
	TAGCAAAGGCGATTAGTCCTC	-45.30	AT4G31730	-40.71
	TTGATTAACGGAGGTTGACGT	-42.25	AT4G31730	-41.78
	TTGATTAACGGAGGTTGACTT	-40.50	AT4G31730	-38.63
	TTACCCTCGTGACAACCGCAC	-48.85	AT4G31730	-42.02
	TTACCCTCGTGACACCCACAA	-47.92	AT4G31730	-39.58
D	TGTCGCCAGATACGTCCGCAA	-49.28	AT4G31730	-37.95
	TAAGGCGATTAGTCCTAGCAT	-43.84	AT4G31730	-42.50

Supplemental Figure S4: Screenshots of WMD3 designer showing amiRNA candidates for each target gene in this study. Red letters on the left indicate corresponding amiRNAs for each target. **(A)** and **(B)**, amiRNAs for silencing *LOG2*. **(C)** amiRNAs for silencing *MIPS1*. **(D)** amiRNAs for silencing *SnRK1.1*. **(E)** amiRNAs for silencing *GDU1*.

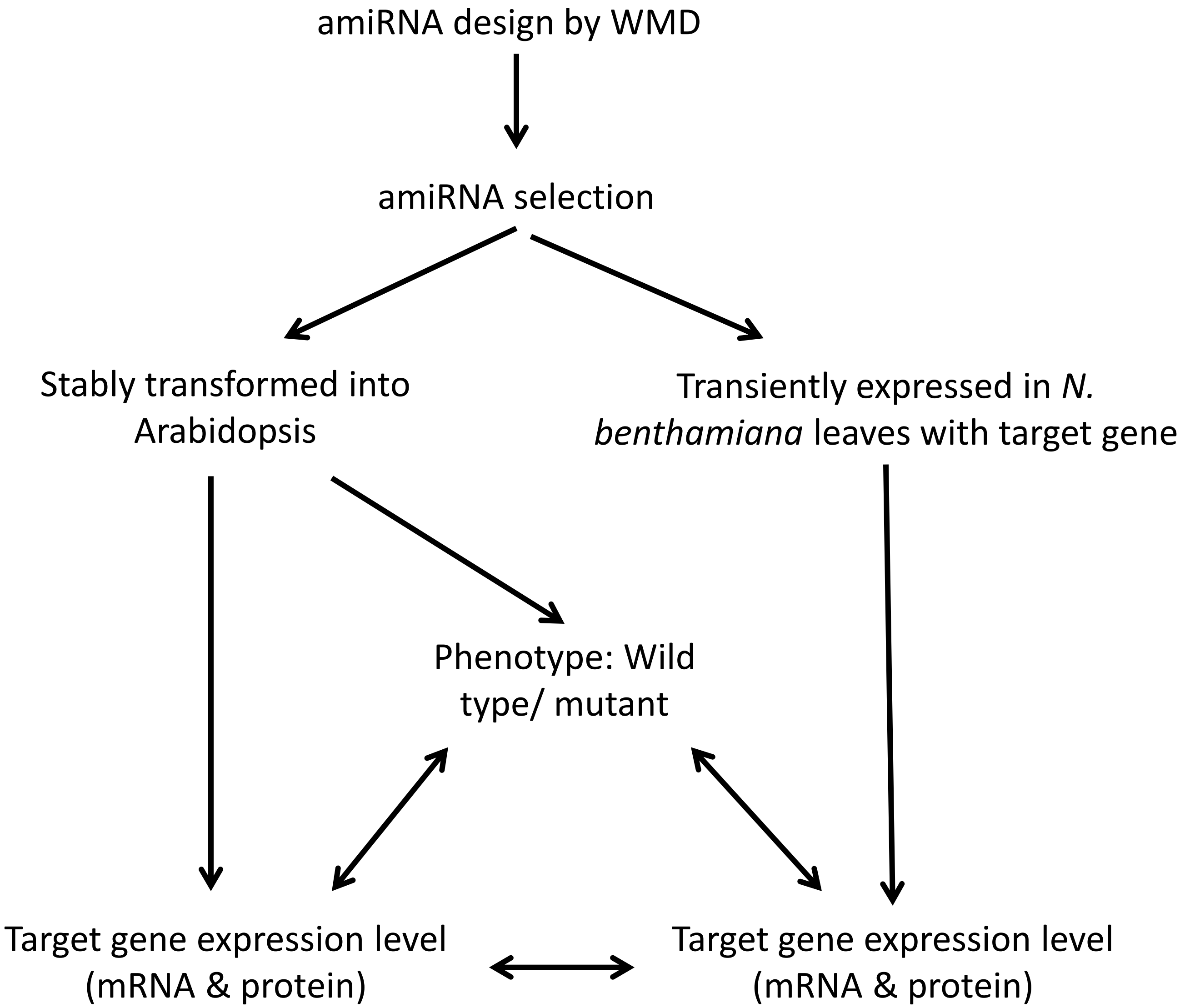

Supplemental Figure S5: Workflow of the study.

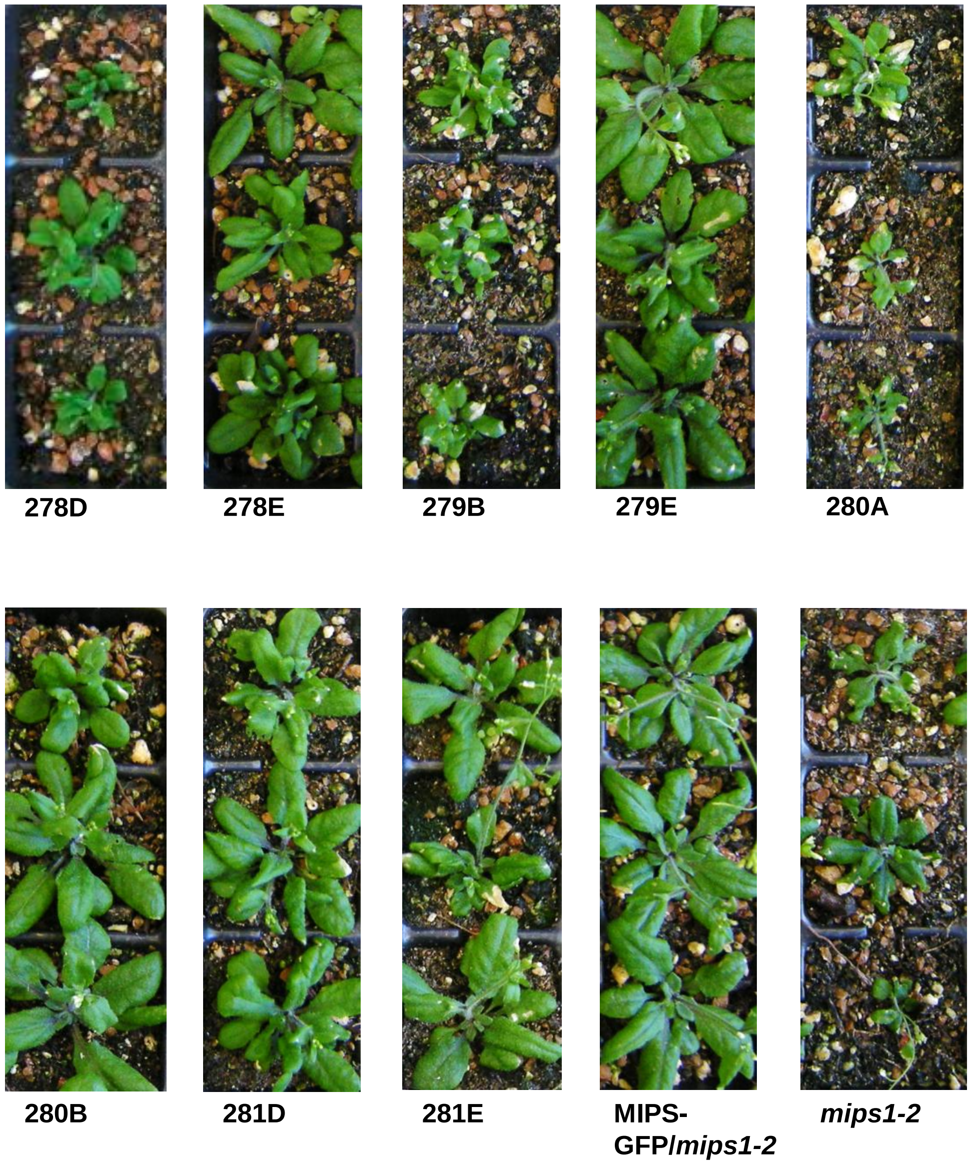

Supplemental Figure S6: Phenotype of one month-old Arabidopsis *mips1-2,* MIPS1-GFP/*mips1-2 and* MIPS1-GFP/*mips1-2* expressing amiRNA^{MIPS1}. Line numbers are the same as Figure 2A.
